# The development and validation of a revised version of the Medical Outcomes Study Sleep Scale (MOS Sleep-R)

**DOI:** 10.1186/s41687-021-00311-3

**Published:** 2021-05-19

**Authors:** Aaron Yarlas, Michelle K. White, Danielle G. St. Pierre, Jakob B. Bjorner

**Affiliations:** 1 QualityMetric, 1301 Atwood Ave, Suite 216E, Johnston, RI USA; 2 Department of Public Health, University of Copenhagen, Gothersgade 160, DK-1123 Copenhagen, Denmark; 3 The Danish National Research Centre for the Working Environment, Lerso Park Alle 105, DK-2100 Copenhagen, Denmark

**Keywords:** Psychometric validation, Sleep, Sleep disturbance, Somnolence, PRO development

## Abstract

**Background:**

The 12-item Medical Outcomes Study Sleep Scale (MOS Sleep Scale) has been used to capture patient-reported sleep problems in hundreds of studies. A revised version of the MOS Sleep Scale (MOS Sleep-R) was developed that uses simplified response sets, provides interpretable norm-based scoring, and has two recall versions (one-week or four-week). The objective of this study was to evaluate the psychometric properties (reliability and construct validity) of the MOS Sleep-R using data from a representative sample of U.S. adults.

**Methods:**

Standardization of raw scores into norm-based *T*-scores (mean = 50, standard deviation = 10) was based on data from a 2009 U.S. internet-based general population survey. The internal consistency reliability of multi-item subscales and global sleep problems indices for both one-week and four-week recall forms of the MOS Sleep-R were examined using Cronbach’s alphas and inter-item correlations. Construct validity was tested by comparing item-scale correlations between items within subscales with item-scale correlations across subscales. Scale-level convergent validity was tested using correlations with measures including generic health-related quality of life (i.e., SF-36v2) and other relevant outcomes (e.g., job performance, number of days in bed due to illness or injury, happiness/satisfaction with life, frequency of stress/pressure in daily life, the impact of stress/pressure on health, and overall health).

**Results:**

The one-week and four-week recall forms of the MOS Sleep-R were completed by 2045 and 2033 respondents, respectively. The psychometric properties of the one-week and four-week forms were similar. All multi-item subscales and global index scores showed adequate internal consistency reliability (all Cronbach’s alpha > 0.75). Patterns of inter-item and item-scale correlations support the scaling assumptions of the MOS Sleep-R. Patterns of correlations between MOS Sleep-R scores with criterion measures of health-related quality of life and other outcomes indicated adequate construct validity.

**Conclusions:**

The MOS Sleep-R introduces a number of revisions to the original survey, including simplified response sets, the introduction of a one-week recall form, and norm-based scoring that enhances interpretability of scores. Both the one-week and four-week recall period forms of the MOS Sleep-R demonstrated good internal consistency reliability and construct validity in a U.S. general population sample.

## Introduction

Sleep is a crucial biological function, strongly tied to physical and mental health outcomes. Optimal sleep is linked to tissue and muscle reparation and growth [1–5], release of growth hormones [6], and memory consolidation [7, 8]. Conversely, disordered or shortened sleep is associated with increased risk for many health consequences including cardiovascular disease [9, 10], inflammation [11], obesity [12], diabetes [13], cognitive impairment [14, 15], injury [16, 17], and mortality [18, 19].

Many people do not get enough sleep [20]. Seven to 9 h of sleep are recommended for adults [21], but approximately 30% of Americans report consistently getting less than 6 h of sleep per night [22]. Self-reported data indicate that 25–40% of people experience poor sleep quality or symptoms of insomnia [23–25]. Given the high rates of insufficient and low quality sleep, it is important to accurately measure and interpret aspects of sleep in both clinical practice and in research.

Sleep can be quantified in a variety of ways. Polysomnography (PSG) [26] is a sleep measure assessing multiple body functions including heart rate, muscle tone, and brain activity that can be scored by stages (e.g., rapid eye movement [REM] sleep). PSG is considered the gold standard for measuring sleep accurately and objectively [27]. Technological devices such as smartwatches (e.g., actiwatches, exercise trackers) can also be used in sleep research [28].

PSG and other sleep measuring devices are unable to thoroughly characterize the impact of sleep problems on an individual. For example, devices cannot describe daytime sleepiness that occurs after a night of poor or insufficient sleep. Patient-reported outcome (PRO) measures can supplement sleep measuring technology or can be used independently and are useful for measuring sleep quality and poor sleep. There are several commonly used PROs focused on aspects of sleep including the Pittsburgh Sleep Quality Index (PSQI) [29], which captures sleep quality, latency, quantity, efficiency, and disturbance, in addition to somnolence and use of sleep medication; the Epworth Sleepiness Scale (ESS) [30], which captures the likelihood of falling asleep when engaged in various daily activities; the Patient-Reported Outcomes Information System (PROMIS) Sleep Disturbance Scale [31], which focuses on the frequency of disturbances during sleep; and the Medical Outcomes Study Sleep Scale (MOS Sleep Scale) [32].

The 12-item MOS Sleep Scale was developed as part of the Medical Outcomes Study [33], a 4 year longitudinal observational study. The MOS Sleep Scale measures sleep quality and problems over the previous four-week period. The original scale captures essential sleep concepts for general populations and for chronic condition groups (e.g., restless leg syndrome, neuropathic pain); it is considered to have good psychometric properties [34, 35], including reliability and construct validity [36]. While item selection and wording did not incorporate input from patients, as was later recommended by the United States Food and Drug Administration (FDA) for ensuring content validity [37], a subsequent cognitive debriefing study, based on interviews with 19 patients with fibromyalgia, reported that patients generally found the items on the MOS Sleep Scale to be appropriate and relevant, capturing all of their sleep-related symptoms [38]. But, patients did recommend some modifications to the MOS Sleep Scale for general use. Based on this feedback, and ongoing use of the MOS Sleep Scale by the developers, three key areas for improvement were identified.

First, the original scale asks respondents to consider their past 4 weeks of sleep when answering the questions; some research areas require sleep measures with a shorter recall period. Utilization of acute recall versions of other scales, including the SF-36 Health Survey, has been supported in previous work [39].

Second, the majority of scale items (10 of 12) use a 6-point scale, with the response options: ‘all of the time,’ ‘most of the time,’ ‘a good bit of the time,’ ‘some of the time,’ ‘a little of the time,’ and ‘none of the time.’ The response option ‘a good bit of the time’ failed to support assumptions of ordinality: respondents reported being unsure of whether the order of this choice falls naturally between its adjacent responses [40]. In addition, the phrasing of this response option had poor translatability, which may account for inconsistencies in the frequency with which respondents chose this option across different translations [40]. Further, studies using item response theory (IRT) modeling to examine other scales have found that the elimination of this response category resulted in little or no loss of item information [41].

Third, some items and subscales are coded such that a higher score is indicative of a more positive sleep outcome (e.g., higher sleep quality), while a higher score on other subscales indicate more negative sleep outcomes (e.g., more sleep disturbance). This leads to a less intuitive interpretation of scores. Finally, the scoring of subscales and global indices on a 0 to 100 scale can be difficult to interpret. For example, would a score of 40 on the somnolence subscale be considered evidence of high or moderate somnolence? Without reference values, this question cannot be answered. Conversion of 0 to 100 scores into *T*-scores (mean = 50, standard deviation [SD] = 10), standardized to the general population, such as has been done for other versions of the MOS Sleep Scale [36], would provide general reference values to help interpretation of scores. Using *T*-scores, a score of 40 would be interpreted as poor, being one SD below the general population normative value.

The MOS Sleep-R was developed to address these limitations. The aim of the present study is to evaluate the psychometric properties of the standard and acute versions of the MOS Sleep-R within a nationally representative sample of adults.

## Methods

### Study design and sample

The data used for this validation were from a subset of the sample in a 2009 internet-based U.S. general population survey conducted by QualityMetric (QM) for the purpose of updating norms for scoring and interpretation of several PROs, including the MOS Sleep-R [42]. The normative sample was recruited from among panelists within KnowledgePanel®, a national address-based probability sample that is estimated to reach 97% of the U.S. non-institutionalized adult population [43], who provided informed consent to participate in this survey, and who received an honorarium for participating. Older panelists were oversampled to ensure adequate inclusion of those with chronic health conditions and to better reflect clinical trial populations for whom this instrument is expected to be most commonly used. Limitations regarding the representativeness of this sampling approach, including response bias of panel members, have been described elsewhere [44]. Respondents in this sample were randomly assigned (with no quotas or stratifications enforced) to one of four surveys, two of which included the MOS Sleep-R. One of these two surveys included the MOS Sleep-R with a one-week recall period (acute form), while the other included the MOS Sleep-R with a four-week recall period (standard form). Each survey also included a number of other PROs, such as the SF-36v2® Health Survey (SF-36v2; a measure of health-related quality of life [HRQoL]); the revised version of the MOS cognitive functioning scale (MOS Cog-R); a checklist to indicate diagnostic history for 40 possible chronic conditions; demographic items; and numerous criterion measures assessing work performance, psychological distress and well-being, medical resource use, and lifestyle behaviors. Recall periods of the SF-36v2 and criterion measures matched that used for the MOS Sleep-R within each survey. A more detailed description of the 2009 QM survey study can be found in the MOS Sleep-R user manual [42] and the SF-36v2 user manual [45].

### Measures

#### Revised MOS sleep scale (MOS sleep-R)

The MOS Sleep-R is a 12-item scale with recall periods of either 4 weeks (standard form) or 1 week (acute form). Following the structure of the original MOS Sleep Scale [36], responses to items afford calculation of 6 subscales related to: sleep disturbances (difficulties in initiating or maintaining sleep; 4 items), snoring (1 item), waking up with shortness of breath or a headache (1 item), adequacy (perceived sufficiency of sleep quality and quantity; 2 items), somnolence (daytime sleepiness and urge to nap; 3 items), and sleep quantity (1 item) (Table 1). Sleep quantity can either be scored continuously (0–24 h), or as a dichotomous variable: optimal (7–9 h, inclusive; coded as ‘1’) vs. non optimal (< 7 or > 9 h; coded as ‘0’) [21].

**Table 1:** MOS Sleep-R items

Item number	Item content [Stem: over the past 1 (4) week(s) …]	Parent subscale	Smallest-Largest possible response value
1^a^	How long did it usually take for you to fall asleep?	Disturbance	1-5^c^
2	On the average, how many hours did you sleep each night?	Quantity	0–24
3^a^	How often did you feel that your sleep was not quiet?	Disturbance	1-5^d^
4^a, b^	How often did you get enough sleep to feel rested upon waking in the morning?	Adequacy	1-5^d^
5^a, b^	How often did you awaken short of breath or with a headache?	Shortness of breath/headache	1-5^d^
6^a^	How often did you feel drowsy or sleepy during the day?	Somnolence	1-5^d^
7^a, b^	How often did you have trouble falling asleep?	Disturbance	1-5^d^
8^a, b^	How often did you awaken during your sleep time and have trouble falling asleep again?	Disturbance	1-5^d^
9^a, b^	How often did you have trouble staying awake during the day?	Somnolence	1-5^d^
10	How often did you snore during your sleep?	Snoring	1-5^d^
11	How often did you take naps during the day?	Somnolence	1-5^d^
12^a, b^	How often did you get the amount of sleep you needed?	Adequacy	1-5^d^

^a^ Item included in the scoring of the 9-item Sleep Problem Index (SPI-II)

^b^ Item included in the scoring of the 6-item Sleep Problem Index (SPI-I)

^c^ Response options are as follows: 1 = All of the time; 2 = Most of the time; 3 = Some of the time; 4 = A little of the time; 5 = None of the time

^d^ Response options are as follows: 1 = 0–15 min; 2 = 16–30 min; 3 = 31–45 min; 4 = 46–60 min; 5= > 60 min

Responses to items also enable calculation of two global index measures of sleep quality and problems: sleep problem index I (SPI-I [6 items]) and sleep problem index II (SPI-II [9 items]). Each index provides a single score that can be interpreted as a general summary of the extent and severity of a respondent’s sleep problems [36].

The MOS Sleep-R kept the content of the MOS Sleep items, but made changes to response options and scoring. First, for the 10 of 12 items that are coded as the frequency of an event (see Table 1), the number of response options was reduced from 6 to 5, with the response option ‘a good bit of the time’ omitted.

Second, the scoring of subscales and indices on a scale from 0 to 100 has been supplemented with norm-based scoring using the normative data from the QM U.S. general population survey. Except for the quantity subscale, all MOS Sleep-R scores are expressed as *T*-scores with the U.S. general population having a mean score of 50 and a SD of 10. The quantity subscale, which is based on a single item asking respondents to report their average number of hours slept per night over the recall period, can be transformed into the optimal quantity subscale. This subscale dichotomizes responses to this item into a score of 1, indicating optimal quantity (a response that is ≥7 and ≤ 9) or 0, indicating non-optimal quantity (all other responses). In all psychometric analyses conducted here, the binary optimal quantity subscale was used. Note that neither raw responses to this item nor the binary coded subscale based on the response contribute to the scoring of either SPI-I or SPI-II.

Third, the direction of scoring for the original scales was subscale specific (i.e., higher scores indicated better sleep outcomes on some subscales, but worse sleep outcomes on others). The scores in the revised scales are interpreted with a single directionality, such that higher scores indicate better sleep for all subscales and both global indices.

#### Criterion measures

A number of scales and individual ad hoc items included in the QM 2009 normative survey were treated as criterion measures in the current survey. One criterion measure was the SF-36v2, which is a 36-item measure of HRQoL [45]. The SF-36v2 is available with both four-week (standard) and one-week (acute) recall periods and measures 8 domains of patients’ well-being and functioning, including physical functioning (PF), role limitations due to physical health problems (RP), bodily pain (BP), general health (GH), vitality (VT), social functioning (SF), role limitations due to emotional health problems (RE), and mental health (MH). Scores on these 8 subscales can be weighted and combined to produce two component summary measures; one for physical HRQoL (Physical Component Summary [PCS]) and one for mental HRQoL (Mental Component Summary [MCS]). All SF-36v2 domains are expressed as norm-based *T*-scores using the data from QM’s 2009 U.S. general population survey, with higher scores indicating better HRQoL. We used the one-week SF-36v2 for comparisons with the one-week version of MOS Sleep-R and the four-week SF-36v2 for comparisons with the four-week version of MOS Sleep-R.

Other criterion measures used in this analysis included ad hoc items measuring self-ratings of job performance, happiness/satisfaction, and overall health, which were all measured on a scale from 0 to 100 with higher numbers indicating better outcomes; and the impact of stress/pressure on health, also measured on a scale from 0 to 100 but with higher numbers indicating a worse outcome (i.e., larger impact). Criterion measures also included the number of days in bed due to illness or injury, and the frequency of stress/pressure in daily life during the same recall-period as was used for the MOS Sleep-R (i.e., the last week or last 4 weeks), and the number of chronic conditions that the respondent endorsed on a checklist.

### Statistical analysis

Data from all respondents in the QM 2009 normative survey who were administered either the standard or the acute form of MOS Sleep-R were included in this analysis. All validation analyses were conducted separately for each form. Analyses were conducted using SPSS v23.0 and SAS v9.4.

Scaling assumptions of the MOS Sleep-R were examined using multiple techniques. First, we assessed the internal consistency reliability of each of the 3 multiple-item subscales (i.e., disturbance, somnolence, and adequacy) and the 2 global indices. Cronbach’s alpha was calculated for items within each of these subscales and indices; sufficient reliability was determined using the conventional threshold of 0.70 [46].

Second, stability of all subscales and both indices was examined based on scores from a subset of 90 respondents in the QM 2009 normative survey who were administered the MOS Sleep-R twice, with the standard and acute forms completed by 45 subjects each. Time between standard form administrations ranged from 80 to 123 days (mean = 106.0 days, SD = 5.9 days), while time between acute form administrations ranged from 80 to 121 days (mean = 105.9 days, SD = 6.4 days) [42]. Stability of scores over time for each subscale and index across the two assessments was evaluated using intraclass correlation coefficients (ICCs) calculated using McGraw and Wong’s Case 3 (A,1) model [47], which is a two-way mixed-effect model with interaction for the absolute agreement for a single measurement that has been recommended as the preferred model for assessing stability of a repeated PRO measure [48, 49]. An ICC value ≥0.70 has been suggested as indicating adequate stability [50].

Third, as part of evaluating construct validity, we tested the assumption of item-subscale convergent validity for each multi-item subscale (and both indices) of the MOS Sleep-R [51, 52]. This assumption holds that each item which contributes to the scoring of a multi-item subscale (or index) should be sufficiently associated with that subscale, by calculating whether each component item within a multi-item subscale correlated at least moderately with its parent subscale (or index). To accomplish this, the magnitude of the Pearson correlation coefficient between each item on the disturbance, somnolence, and adequacy subscales, and its corrected-parent subscale (i.e., the correlation between the item and its parent subscale when the subscale is calculated using only the remaining component items) or index was compared to a threshold value of 0.40, which can be interpreted as support for convergent validity [50]. Means of correlations between items and their corrected-parent subscale for each multi-item domain were computed using Fisher’s r-to-z approach [53].

Fourth, we tested the assumption of item-subscale discriminant validity for each multi-item subscale of the MOS Sleep-R. This assumption holds that each item which contributes to the scoring of a multi-item subscale should be more highly correlated with that parent subscale than with any of the other subscales [51, 52]. This was achieved by descriptively comparing the magnitudes of Pearson correlations (or, in the case of the binary optimal quantity subscale, polychoric correlations, which are more appropriate for evaluating associations with an ordinal variable) between items and their corrected-parent subscale with the magnitudes of correlations between items and the remaining 5 subscales.

Fifth, scale-level convergent validity of the MOS Sleep-R subscale scores (which is the degree to which scores correspond to conceptually related constructs) was examined using correlations. Magnitudes of correlation coefficients were interpreted following Cohen’s guidelines, such that correlations of 0.1, 0.3, and 0.5 represented small, moderate, and large correlations, respectively [54]. Pearson correlations (or polychoric correlations for the optimal quantity subscale, comprised of one item) were calculated between subscale and the two SPI indices on the standard and acute forms of the MOS Sleep-R, and the corresponding form of the SF-36v2. Based on previous work examining the association between sleep quality and HRQoL, which has found a stronger correspondence of sleep with mental aspects of HRQoL than with physical aspects [55–57], along with the well-established evidence for associations between sleep problems and pain [58], we predicted that with the exception of the BP domain, MOS Sleep-R scores would generally show higher correlations (based on descriptive comparisons) with mental-based subscales of the SF-36v2 (VT, SF, RE, and MH) and with MCS than with physical-based subscales and with PCS. Spearman rank-order correlations (or polychoric correlations for the optimal quantity subscale) were calculated for scores on each MOS Sleep-R subscale and index with scores on the selected criterion measures described in the previous section. In general, positive correlations were expected between MOS Sleep-R scores and perceptions of job performance and overall health, while negative correlations were expected between MOS Sleep-R scores and the number of days in bed due to illness or injury, happiness/satisfaction with life (with higher scores indicating less happiness/satisfaction), frequency of stress/pressure in daily life, the impact of stress/pressure on health, and the number of chronic conditions endorsed. For the correlational analyses described above (i.e., correlations between MOS Sleep-R items and subscales, inter-scale correlations among MOS Sleep-R subscale scores, correlations between MOS Sleep-R and SF-36v2 scores, and correlations between MOS Sleep-R and other criterion measures), we performed sensitivity analyses using polychoric correlations. A non-trivial difference in magnitude of coefficients between standard Pearson or Spearman correlations and polychoric correlations would suggest that the use of Pearson or Spearman correlations was not appropriate. For the first three of these sensitivity analyses, polychoric correlations were similar to Pearson correlations, and so Pearson correlations were reported for those analyses (with the exception of correlations involving the optimal quantity subscale). For the third sensitivity analysis (correlations between MOS Sleep-R and other criterion measures), differences between Spearman correlations and polychoric correlations were observed. Thus, results from polychoric correlations were reported for this analysis.

Sixth, known-groups validity of MOS Sleep-R subscales (with the exception of sleep quantity) and index scores was examined by comparing scores between respondents who did and did not self-report having sleep apnea, and between respondents who did or did not self-report health conditions known to be associated with sleep problems: rheumatoid arthritis [RA] [59], and osteoarthritis [OA] [60]. For each health condition, statistically significant differences in mean MOS Sleep-R scores between respondents reporting having or not having the condition were tested using independent-samples t-tests. Cohen’s *d* was used to estimate magnitude of standardized differences in means between groups; values were interpreted according to Cohen’s published guidelines (*d* = 0.2, small effect; *d* = 0.5, medium effect; *d* = 0.8, large effect) [54].

## Results

### Sample characteristics and descriptive statistics

A total of 4098 respondents completed the MOS Sleep-R in the QM 2009 normative survey, with 2037 completing the standard recall form and 2061 completing the acute recall form. The completion rate across all surveys was 66.0% [45]. Demographic characteristics of the sample, presented in Table 2, were similar across both forms. Both genders were close to equally represented, while age was older than that of the U.S. general population (mean age was 50.8 for the standard form group and 50.6 for the acute form group) due to deliberate oversampling of older panelists.

**Table 2:** Patient characteristics of validation sample

	*Standard Form (N = 2037)*		*Acute Form (N = 2061)*	
	n	%	n	%
**Gender**				
Male	1019	50	1011	49
Female	1018	50	1050	51
**Age**				
18–24	167	8	163	8
25–34	250	12	286	14
35–44	337	17	354	17
45–54	346	17	337	16
55–64	396	19	378	18
65–74	396	19	378	18
75+	145	7	165	8
**Employment Status**				
Working	1076	53	1096	53
Retired/disabled	683	34	666	32
Temporarily laid off/looking for work	144	7	155	8
Not working/other	134	7	144	7
**Ethnicity/Race**				
White	1647	84	1638	82
Black or African American	187	10	202	10
American Indian or Alaska Native	18	1	21	1
Asian	29	2	43	2
Native Hawaiian/Pacific Islander	3	0	5	0
Multiracial	82	4	91	5
**Education**				
Less than high school	168	8	174	8
High school	613	30	634	31
Some college/other training	641	32	611	30
Bachelor’s degree or higher	615	30	642	31

Percentages may not add to 100 across categories due to rounding

Means and SDs for raw item scores from each form are shown in Table 3. Values of item scores showed little variation between the two forms; all scores were within 0.10 points (on 5-point response scales) on all items. Average sleep from both forms was slightly less than 7 h, which is consistent with findings from other nationally representative surveys measuring sleep [61].

**Table 3:** MOS Sleep-R standard and acute form item score means and standard deviations

Item Number	Abbreviated Content	Parent subscale	*Standard Form*			*Acute Form*		
			n	Mean	SD	n	Mean	SD
1^a^	Time to fall asleep	Disturbance	2020	3.86	1.29	2015	3.90	1.31
2	Average hours of sleep	Quantity	2019	6.87	1.88	2025	6.89	2.07
2^b^	Sleep quantity (optimal/non-optimal)	Optimal Quantity	2008	0.47	0.50	2008	0.52	0.50
3^a^	Sleep was not quiet	Disturbance	2017	3.61	1.16	2020	3.64	1.23
4^a, c^	Enough sleep to feel rested on waking	Adequacy	2018	3.23	1.08	2020	3.19	1.18
5^a, c^	Awaken short of breath or with a headache	Shortness of breath/headache	2009	4.56	0.85	2021	4.55	0.89
6^a^	Feel drowsy/sleepy during the day	Somnolence	2019	3.42	1.05	2022	3.44	1.16
7^a, c^	Trouble falling asleep	Disturbance	2021	3.77	1.22	2024	3.83	1.29
8^a, c^	Trouble falling asleep again after awakening	Disturbance	2012	3.89	1.11	2024	3.96	1.18
9^a, c^	Trouble staying awake during the day	Somnolence	2016	4.14	0.95	2023	4.14	1.02
10	Snoring	Snoring	1992	3.68	1.32	2002	3.78	1.26
11	Take naps during the day	Somnolence	2013	3.90	1.14	2022	3.98	1.14
12^a, c^	Get needed amount of sleep	Adequacy	2018	3.20	1.15	2021	3.21	1.24

^a^ Item included in the scoring of the 9-item Sleep Problem Index (SPI-II)

^b^ Scored as optimal (7–9 h, inclusive) or non-optimal (< 7 or > 9 h), where optimal = 1 and non-optimal = 0

^c^ Item included in the scoring of the 6-item Sleep Problem Index (SPI-I)

### Reliability and construct validity

Findings from assessments of internal consistency for the 3 multi-item subscales and the 2 SPI indices for standard and acute forms of the MOS Sleep-R are reported in Table 4. Cronbach’s alphas for all multi-item subscales and indices of both forms exceeded 0.70, with the majority exceeding 0.80, indicating acceptable reliability.

**Table 4:** Summary of internal consistency, stability, and construct validity for MOS Sleep-R standard and acute form subscales and global index measures

Form	Subscale/Index	Number of items	Stability (ICC)	Internal consistency (Cronbach’s alpha)	Mean item-corrected parent subscale correlation within subscale (minimum-maximum)	Mean item-non-parent subscale correlation across subscales (minimum-maximum)	Scaling success^**a**^	Success rate (%)^**b**^
Standard	Disturbance	4	0.81	0.85	0.71 (0.61–0.82)	0.36 (0.10–0.55)	20	100
	Adequacy	2	0.68	0.84	0.72 (0.72–0.72)	0.39 (0.15–0.57)	10	100
	Somnolence	3	0.81	0.76	0.61 (0.49–0.71)	0.33 (0.18–0.56)	15	100
	Snoring	1	0.85	–	–	0.22 (0.19–0.27)	–	–
	SOBH	1	0.33	–	–	0.32 (0.22–0.40)	–	–
	Optimal quantity	1	0.34	–	–	0.19 (0.04–0.41)	–	–
	SPI-I	6	0.75	0.83	0.61 (0.41–0.69)	–	–	–
	SPI-II	9	0.81	0.88	0.63 (0.43–0.75)	–	–	–
Acute	Disturbance	4	0.78	0.86	0.72 (0.62–0.82)	0.41 (0.16–0.56)	20	100
	Adequacy	2	0.70	0.85	0.74 (0.74–0.74)	0.44 (0.20–0.59)	10	100
	Somnolence	3	0.75	0.78	0.64 (0.51–0.75)	0.37 (0.18–0.63)	14	93
	Snoring	1	0.83	–	–	0.24 (0.20–0.28)	–	–
	SOBH	1	0.69	–	–	0.39 (0.23–0.48)	–	–
	Optimal quantity	1	0.71	–	–	0.21 (0.07–0.32)	–	–
	SPI-I	6	0.82	0.85	0.64 (0.50–0.69)	–	–	–
	SPI-II	9	0.82	0.90	0.67 (0.51–0.76)	–	–	–

^a^ The number of item-subscale correlations across subscales that are descriptively smaller in magnitude than the correlations between each item and its corrected-parent subscale

^b^ The percentage of item-subscale correlations achieving scaling success

– not computable, *ICC* intraclass correlation coefficient, *SOBH* awakening due to shortness of breath or headache, *SPI* sleep problems index

Results for evaluation of stability are also reported in Table 4. For the standard form ICC ≥ 0.70 was found for both indices and 3 subscales – disturbance, somnolence, and snoring – with the adequacy subscale falling just below the cut-off (ICC = 0.68). Both the shortness of breath/headache and the optimal quantity subscales had poor stability for the standard form (both ICC < 0.35). For the acute form, results found ICC ≥ 0.70 for all subscales and indices except the shortness of breath/headache subscale, where ICC = 0.69.

Table 4 also reports results from the examination of the construct validity of the MOS Sleep-R. For both forms, all Pearson correlations between component items and their corrected-parent subscale or index exceeded the criterion of 0.40. In fact, mean correlations of items for each subscale and index > 0.60, thus providing strong support for the item-level convergent validity of these subscales. On the standard form, for all 3 multi-item subscales (i.e., disturbance, adequacy, and somnolence), all items hand higher correlations with their corrected-parent subscale than with any other subscale. On the acute form, this was again the case for the disturbance and adequacy subscales, and for 14 of 15 correlations for the 3 items on the somnolence subscale, with the only deviation being for the item “How often did you feel drowsy or sleepy during the day,” for which there was a very similar correlation with the adequacy subscale (*r* = 0.63) compared with its own corrected-parent subscale (*r* = 0.62).

Table 5 shows intercorrelations among subscales and between subscales and global indices. The generally low magnitude of the inter-subscale correlations – the only correlations exceeding 0.5 were the correlation between adequacy and disturbance (0.60) for the standard form, and correlations between adequacy and disturbance (0.60), somnolence and disturbance (0.55), and somnolence and adequacy (0.54) for the acute form – indicates that magnitudes of correlations among the subscales are mostly small or moderate, and thus appear to be capturing separate constructs.

**Table 5:** Inter-subscale and index-subscale correlations for MOS Sleep-R standard and acute forms

Form	Subscale/Index	Disturbance	Adequacy	Somnolence	Snoring	SOBH	Optimal Quantity^a^
Standard	Disturbance	–					
	Adequacy	0.60	–				
	Somnolence	0.46	0.48	–			
	Snoring	0.20	0.19	0.27	–		
	SOBH	0.38	0.31	0.40	0.22	–	
	Optimal quantity^a^	0.45	0.46	0.34	0.20	0.26	–
	SPI-I	-^b^	-^b^	-^b^	0.26	-^b^	0.49
	SPI-II	-^b^	-^b^	-^b^	0.26	-^b^	0.50
Acute	Disturbance	–					
	Adequacy	0.63	–				
	Somnolence	0.55	0.54	–			
	Snoring	0.28	0.24	0.27	–		
	SOBH	0.48	0.39	0.44	0.23	–	
	Optimal quantity^a^	0.43	0.50	0.38	0.20	0.39	–
	SPI-I	-^b^	-^b^	-^b^	0.31	-^b^	0.51
	SPI-II	-^b^	-^b^	-^b^	0.31	-^b^	0.51

All coefficients were Pearson correlations except as otherwise specified

*SOBH* awakening due to shortness of breath or headache, *SPI* sleep problems index

^a^ Coefficients were polychoric correlations

^b^ Correlation not calculated because of item overlap

### Scale-level convergent validity

Correlations between MOS Sleep-R scores for the standard and acute forms, and SF-36v2 scores from the corresponding form are presented in Table 6. Both forms showed very similar patterns of inter-scale correlations. In general, small to moderate correlations were observed between scores on the two scales. Among MOS Sleep-R subscales, disturbance and somnolence generally showed the highest correlations with SF-36v2 scores, while snoring and optimal quantity generally showed the smallest correlations.

**Table 6:** Correlation coefficients between MOS Sleep-R and SF-36v2 standard and acute form subscales and summary measures

		SF-36v2 Subscale/Index									
Form	Subscale/Index	PF	RP	BP	GH	VT	SF	RE	MH	PCS	MCS
Standard	Disturbance	0.37	0.39	0.46	0.48	0.53	0.47	0.47	0.53	0.36	0.50
	Adequacy	0.24	0.26	0.37	0.43	0.61	0.37	0.33	0.49	0.26	0.47
	Somnolence	0.44	0.47	0.46	0.51	0.60	0.49	0.44	0.45	0.45	0.45
	Snoring	0.14	0.13	0.21	0.21	0.20	0.16	0.17	0.17	0.16	0.17
	SOBH	0.39	0.41	0.39	0.40	0.41	0.49	0.46	0.45	0.35	0.44
	Optimal quantity^a^	0.30	0.33	0.27	0.31	0.34	0.33	0.27	0.25	0.28	0.22
	SPI-I	0.42	0.44	0.52	0.56	0.68	0.55	0.51	0.61	0.42	0.58
	SPI-II	0.43	0.45	0.52	0.57	0.68	0.56	0.53	0.62	0.43	0.60
Acute	Disturbance	0.41	0.45	0.48	0.54	0.59	0.52	0.48	0.57	0.40	0.53
	Adequacy	0.29	0.32	0.39	0.48	0.66	0.46	0.37	0.59	0.28	0.55
	Somnolence	0.44	0.49	0.44	0.56	0.64	0.54	0.47	0.53	0.43	0.52
	Snoring	0.17	0.18	0.20	0.23	0.22	0.16	0.14	0.19	0.19	0.15
	SOBH	0.36	0.41	0.39	0.44	0.44	0.47	0.42	0.44	0.35	0.43
	Optimal quantity^a^	0.25	0.30	0.30	0.32	0.32	0.38	0.34	0.32	0.23	0.27
	SPI-I	0.43	0.49	0.51	0.61	0.72	0.60	0.52	0.66	0.42	0.63
	SPI-II	0.45	0.49	0.53	0.61	0.73	0.60	0.53	0.67	0.43	0.64

All coefficients were Pearson correlations except as otherwise specified

*PF* physical functioning, *RP* role physical, *BP* bodily pain, *GH* general health, *VT* vitality, *SF* social functioning, *RE* role emotional, *MH* mental health, *PCS* physical component summary, *MCS* mental component summary, *SOBH* awakening due to shortness of breath or headache, *SPI* sleep problems index

^a^ Coefficients were polychoric correlations

Among SF-36v2 domains, VT, SF, and MH generally showed the highest correlations with MOS Sleep-R scores, while PF and RP generally showed the smallest correlations. This trend towards higher correlations between MOS Sleep-R scores and mental-based domains is clearly indicated by the higher correlations among indices and summary measures, with correlations between SPI-I/SPI-II and PCS scores ranging from 0.42 to 0.43 across forms, and correlations between SPI-I/SPI-II and MCS scores ranging from 0.58 to 0.64. Overall, the sleep problem indices tended to have the largest correlations with the SF-36v2 mental domains.

Polychoric correlations between MOS Sleep-R scores and other criterion measures are shown in Table 7. Both forms showed very similar patterns of correlations with these criterion measures. Correlations between MOS Sleep-R scores and all criterion measures were in the hypothesized direction: positive correlations were observed for the 3 positively-worded items (ratings of job performance, happiness, and overall health), while negative correlations were observed for the remaining negatively-worded items. Correlations with the number of current chronic conditions, which were negative across all subscales, were highest for disturbance, somnolence, and shortness of breath/headache subscales. The multi-item scales generally show stronger correlations than the single-item measures – presumably due to higher reliability of the multi-item scales.

**Table 7:** Correlation coefficients between MOS Sleep-R scores and convergent validity measures

		Convergent validity measures						
Form	Subscale/Index	Job performance	Days in bed due to illness/injury	Happiness/ satisfaction	Frequency of feeling stress/pressure	Impact of stress/pressure on health	Overall health	Number of current chronic conditions
Standard	Disturbance	0.25	−0.53	0.50	− 0.41	− 0.50	0.44	− 0.42
	Adequacy	0.24	−0.42	0.46	−0.48	−0.49	0.42	−0.34
	Somnolence	0.27	−0.47	0.42	−0.36	−0.43	0.45	−0.45
	Snoring	0.09	−0.15	0.15	−0.15	−0.17	0.19	−0.22
	SOBH	0.25	−0.51	0.44	−0.37	−0.52	0.44	−0.41
	Optimal quantity	0.13	−0.29	0.28	−0.22	−0.30	0.25	−0.27
	SPI-I	0.31	−0.57	0.55	−0.50	−0.58	0.53	−0.47
	SPI-II	0.30	−0.58	0.56	−0.51	−0.58	0.52	−0.49
Acute	Disturbance	0.25	−0.48	0.52	−0.45	−0.49	0.45	−0.47
	Adequacy	0.24	−0.43	0.52	−0.52	−0.51	0.46	−0.35
	Somnolence	0.22	−0.46	0.44	−0.44	−0.49	0.44	−0.44
	Snoring	0.11	−0.22	0.21	−0.20	−0.22	0.22	−0.18
	SOBH	0.17	−0.54	0.43	−0.47	−0.54	0.48	−0.54
	Optimal quantity	0.12	−0.28	0.31	−0.29	−0.32	0.30	−0.24
	SPI-I	0.28	−0.54	0.58	−0.55	−0.59	0.52	−0.49
	SPI-II	0.28	−0.54	0.59	−0.56	−0.59	0.53	−0.50

All coefficients were Polychoric correlations

*SOBH* awakening due to shortness of breath or headache, *SPI* sleep problems index

Among MOS Sleep-R subscales, disturbance, adequacy, and somnolence generally showed the highest correlations with criterion measures, while snoring and optimal quantity generally showed the smallest correlations. Among criterion measures, those measuring psychological constructs (happiness/satisfaction, stress/pressure) showed the highest correlations with MOS Sleep-R scores, while those measuring perceived job performance and days in bed due to illness or injury showed the smallest correlations.

### Known-groups validity

Comparisons of mean MOS Sleep-R scores between patients with or without sleep apnea, RA, and OA are presented in Table 8. Mean scores for all subscale and SPI-I/SPI-II were statistically significantly worse for respondents self-reporting sleep apnea than for those not self-reporting sleep apnea (all *p* < 0.001 for both standard and acute forms), for respondents self-reporting RA than for those not self-reporting RA (all *p* < 0.001 for standard form; all *p* < 0.05 for acute form), and for respondents self-reporting OA than for those not self-reporting OA (all *p* < 0.001 for both standard and acute forms). Effect sizes for subscale differences were generally medium-sized between sleep apnea groups (Cohen *d*’s ranging from 0.46 to 0.90 for the standard form and 0.43 to 0.79 for the acute form), and generally small to medium-sized between RA groups (0.28 to 0.65 for the standard form and 0.18 to 0.75 for the acute form) and between OA groups (0.24 to 0.65 for the standard form and 0.26 to 0.63 for the acute form). Deficits on the somnolence subscale were uniformly largest for each health condition; impacts on snoring were smallest for OA and RA groups.

**Table 8:** Differences in mean MOS Sleep-R scores as a function of self-reported health condition

		Sleep Apnea^**a**^				Rheumatoid Arthritis^**a**^				Osteoarthritis^**a**^			
**Form**	**Subscale/Index**	**Yes (*n*** **= 194)**	**No (*n*** **= 1829)**	***p***	***d***	**Yes (*n*** **= 154)**	**No (*n*** **= 1869)**	***p***	***d***	**Yes (*n*** **= 257)**	**No (*n*** **= 1766)**	***p***	***d***
Standard	Disturbance	44.6 (11.2)	50.6 (9.7)	< 0.001	0.61	44.6 (11.2)	50.5 (9.8)	< 0.001	0.59	45.3 (10.9)	50.7 (9.7)	< 0.001	0.55
	Adequacy	45.9 (10.1)	50.4 (9.9)	< 0.001	0.46	46.4 (10.2)	50.3 (9.9)	< 0.001	0.40	47.4 (10.0)	50.4 (9.9)	< 0.001	0.30
	Somnolence	42.1 (11.2)	50.8 (9.5)	< 0.001	0.90	44.1 (11.1)	50.5 (9.8)	< 0.001	0.65	44.5 (11.0)	50.8 (9.6)	< 0.001	0.65
	Snoring	44.3 (12.2)	50.6 (9.5)	< 0.001	0.64	47.4 (10.6)	50.2 (9.9)	< 0.001	0.28	47.9 (9.8)	50.3 (10.0)	< 0.001	0.24
	SOBH	42.4 (15.3)	50.8 (8.9)	< 0.001	0.87	46.1 (13.0)	50.3 (9.6)	< 0.001	0.43	46.1 (13.2)	50.6 (9.3)	< 0.001	0.45
	SPI-I	42.9 (10.7)	50.8 (9.6)	< 0.001	0.80	44.3 (10.3)	50.5 (9.8)	< 0.001	0.63	45.0 (10.8)	50.7 (9.7)	< 0.001	0.59
	SPI-II	43.1 (10.5)	50.7 (9.7)	< 0.001	0.79	44.3 (10.4)	50.5 (9.8)	< 0.001	0.62	45.0 (10.6)	50.7 (9.7)	< 0.001	0.58
**Form**	**Subscale/Index**	**Yes (*n*** **= 205)**	**No (*n*** **= 1821)**	***p***	***d***	**Yes (*n*** **= 161)**	**No (*n*** **= 1865)**	***p***	***d***	**Yes (*n*** **= 272)**	**No (*n*** **= 1753)**	***p***	***d***
Acute	Disturbance	45.4 (11.2)	50.5 (9.7)	< 0.001	0.52	44.4 (11.7)	50.5 (9.7)	< 0.001	0.61	45.6 (11.5)	50.7 (9.6)	< 0.001	0.52
	Adequacy	46.2 (9.9)	50.4 (9.9)	< 0.001	0.43	48.2 (10.7)	50.2 (9.9)	0.015	0.20	47.6 (10.4)	50.4 (9.9)	< 0.001	0.28
	Somnolence	43.1 (11.9)	50.8 (9.4)	< 0.001	0.79	43.2 (12.5)	50.6 (9.5)	< 0.001	0.75	44.7 (11.7)	50.8 (9.4)	< 0.001	0.63
	Snoring	43.7 (11.0)	50.7 (9.6)	< 0.001	0.72	48.3 (10.7)	50.1 (9.9)	0.031	0.18	47.7 (10.0)	50.3 (10.0)	< 0.001	0.26
	SOBH	45.2 (13.2)	50.5 (9.4)	< 0.001	0.55	44.4 (14.8)	50.5 (9.3)	< 0.001	0.62	46.5 (12.8)	50.5 (9.4)	< 0.001	0.41
	SPI-I	43.9 (11.4)	50.7 (9.6)	< 0.001	0.70	44.4 (12.6)	50.5 (9.6)	< 0.001	0.62	45.7 (11.5)	50.7 (9.6)	< 0.001	0.51
	SPI-II	44.2 (11.3)	50.7 (9.6)	< 0.001	0.66	44.3 (12.4)	50.5 (9.6)	< 0.001	0.63	45.4 (11.4)	50.7 (9.6)	< 0.001	0.54

Data are presented as mean (standard deviation). Sample sizes may vary slightly by subscale/index

*SOBH* awakening due to shortness of breath or headache, *SPI* sleep problems index

^a^ Presence or absence of health conditions were self-reported

## Discussion

Data from a large sample of individuals from the U.S. general population were analyzed to evaluate the psychometric properties of the MOS Sleep-R. The results provide support for good reliability and validity of both the standard and acute recall versions in the general population. Specifically, the instrument demonstrated acceptable reliability, as assessed using both internal consistency and stability. Correlations between component items and their parent subscale, correlations among subscales, and correlations between subscales and external variables provide strong evidence supporting construct validity of the instrument.

Patterns of inter-item correlations between the MOS Sleep-R and a generic measure of HRQoL (the SF-36v2) were in expected directions. As predicted, mental-based domains of the SF-36v2 were more highly correlated with the MOS Sleep-R than physical domains. This reflects findings in the literature that show a strong relationship between mental health and sleep [62–64]. Higher (i.e., better) scores on the MOS Sleep-R were also associated with more positive ratings of job performance, happiness, and overall health. These results provide support for the convergent validity of the MOS Sleep-R. Further, respondents who self-reported sleep apnea and those who self-reported health conditions known to be associated with sleep problems, showed consistently worse scores on all MOS Sleep-R subscales and indices than their counterparts.

The rationale behind most of the changes made to develop the MOS Sleep-R was to improve the usability of the scale (by providing an acute form with a shorter recall period) and to improve the interpretability of scores (by making all scoring unidirectional and norm-based). These changes are unlikely to have made a large impact on the psychometric properties of the measure [65]. It was not an objective of the current study to compare the psychometric properties of the MOS Sleep-R with the original MOS Sleep Scale, or to provide evidence for a claim that the changes made to the MOS Sleep-R lead to improvement in psychometric properties compared to the original scale, which, as mentioned above, has been shown to have good reliability and construct validity. The objective of the current study, then, was to confirm that the newly developed MOS Sleep-R, with improved utility and interpretability compared to the original scale, also showed evidence of good reliability and construct validity. As such, the current study did not include a direct comparison between the MOS Sleep Scale and MOS Sleep-R.

In spite of sleep being an important contributor to overall health and well-being, many people suffer from poor sleep. The MOS Sleep-R yields scores that can be more easily understood than those provided by previous versions. Improvements to this scale allow for interpretation of scores relative to the general population. The MOS Sleep-R norm-based scoring provides a comparison point for any condition that may affect sleep. This could be used to show where a specific group (e.g., patients with restless leg syndrome) fall on the scale relative to the general population, allowing researchers and clinicians to better understand the burden of different conditions on sleep. While some state-of-the-art PRO measures, such as the PROMIS measures [66], use different directions for scoring depending on the domain, results from tests with patients and clinicians suggest that it is easier for them to interpret the scores if higher scores consistently indicates better health [67].

While most correlations among subscales and indices were high, the snoring subscale had small correlations with all other measures and showed the smallest deficits for respondents self-reporting RA or OA. Breathing patterns at night may be difficult to self-report accurately; some respondents may be unaware of the presence or frequency of their own snoring. Low accuracy in reporting could explain the lack of high correlations between this subscale and other sleep constructs captured by the MOS Sleep-R. Snoring is associated with known sleep-related conditions (e.g., sleep apnea, for which deficits observed in this study were medium-sized) and it is therefore important to capture this behavior despite its relative lack of association with the other measured concepts in this instrument.

Results from the evaluation of stability of the MOS Sleep-R should be interpreted with caution. First, the interval between administrations for the 90 patients in the test-retest subsample, which ranged between 80 and 123 days with a mean of 106 days, is much longer than is typically, or optimally, used for this purpose. In addition, we did not assess whether a respondent had experienced actual change in the target construct during this interval, which would have allowed for restricting the test-retest sample to include only respondents with stable sleep behaviors. Each of these factors increases the probability that respondents in the subsample actually experienced change in the measured constructs, which violates the core assumption of repeatability (i.e., the measured construct is unchanged between assessments), and as such underestimates the ‘true’ stability of the MOS Sleep-R. This may explain the poor stability observed for some of the subscales, in particular shortness of breath/headache and optimal quantity subscales for the standard form.

These findings should be interpreted in the light of some additional limitations. This study used a representative sample of the U.S. population but people in other countries may have different sleep experiences and expectations than those in the U.S. For example, daytime napping is an established part of the culture in multiple countries, such as Spain, Italy, and Japan [68], but not in other countries. Thus, a person in the U.S. may be more likely to perceive daytime napping as an indication of somnolence than a person from another country where this behavior is considered ‘normal’. Other work has established global variations in sleep patterns (such as wake times) and sleep problems (such as insomnia) [69, 70]. These differences should be considered when interpreting findings from the MOS Sleep-R for individuals outside the U.S.

Results from the current study were based on a general population sample. Previous research has shown that good psychometric properties observed for scales developed and validated in a general population sample may not hold up when used with clinical samples of patients with considerably worse sleep problems [71]. In a general population sample it would be expected to observe a fairly wide range of sleep quality and problems, with many respondents having very mild or no sleep problems. However, the variability of sleep problems in a clinical sample of patients with a health condition associated with sleep problems would likely be more limited, with possible floor effects. The resultant restriction of range in scores could lead to biased underestimation of correlations among MOS Sleep-R items and subscales, reduce correlations between MOS Sleep-R scores and criterion measures, and reduce differences among subgroups differing on clinical characteristics. As such, it cannot be claimed from the current study data that the MOS Sleep-R would exhibit similar psychometric properties when used within a clinical sample. Future studies administering the MOS Sleep-R in clinical samples of patients with sleep problems could address this issue.

With the exception of a small subsample for which there were two assessments, this study used a cross-sectional sample. Use of longitudinal methods or an intervention study design would allow evaluation of the scale’s responsiveness across multiple time points.

This study did not include additional measures of sleep other than the MOS Sleep-R. Our criterion measures were mostly comprised of second-order outcomes (those that are known to be indirectly related to sleep) rather than actual measures of sleep quality and problems. Future studies in which the MOS Sleep-R is administered alongside other methods of capturing sleep quality and problems, such as sleep-measuring devices or different sleep-related PROs, would provide further evidence for evaluating the convergent validity of this scale.

Finally, the psychometric analyses used in this study was based on classical psychometric techniques. This approach is in line with original work on the MOS Sleep instrument. However, more thorough analyses of construct validity could be performed using confirmatory factor analysis, structural equation models, and IRT models [31]. These approaches could be used in future work. We also encourage qualitative studies, in both general population and clinical samples, to examine the content validity of the MOS Sleep-R.

## Conclusions

The MOS Sleep-R has good psychometric properties in the general U.S. population and is recommended for capturing sleep quality and problems in both clinical trials and practice. Future work should evaluate discriminant validity and responsiveness of the instrument over time.

## Data Availability

Specific data points can be made available upon reasonable request.

## Abbreviations

BP: Bodily Pain
ESS: Epworth Sleepiness Scale
FDA: United States Food and Drug Administration
GH: General Health
HRQoL: Health-related quality of life
ICC: Intraclass correlation coefficients
IRT: Item response theory
MCS: Mental Component Summary
MH: Mental Health
MOS Cog-R: MOS cognitive functioning scale
MOS Sleep: Medical Outcomes Study Sleep Scale
MOS Sleep-R: Medical Outcomes Study Sleep Scale Revised
OA: Osteoarthritis
PCS: Physical Component Summary
PF: Physical Functioning
PRO: Patient-reported outcome
PROMIS: Patient-Reported Outcomes Information System
PSG: Polysomnography
PSQI: Pittsburgh Sleep Quality Index
QM: QualityMetric
RA: Rheumatoid Arthritis
REM: Rapid Eye Movement
RE: Role-Emotional
RP: Role-Physical
SD: Standard deviation
SF: Social Functioning
SPI-I: Sleep problem index I
SPI-II: Sleep problem index II
VT: Vitality
